# *I*mmuno*g*lobuli*N* *i*n the *T*reatment of *E*ncephalitis (IgNiTE): protocol for a multicentre randomised controlled trial

**DOI:** 10.1136/bmjopen-2016-012356

**Published:** 2016-11-03

**Authors:** M A Iro, M Sadarangani, M Absoud, W K Chong, C A Clark, A Easton, V Gray, R Kneen, M Lim, M Pike, T Solomon, A Vincent, L Willis, L-M Yu, A J Pollard

**Affiliations:** 1 Oxford Vaccine Group, Department of Paediatrics, University of Oxford and NIHR Biomedical Research Centre, Oxford University Hospitals NHS Foundation Trust, Oxford, UK; 2 Department of Paediatrics, Oxford University Hospitals NHS Foundation Trust, Oxford, UK; 3 Vaccine Evaluation Center, BC Children's Hospital Research Institute, University of British Columbia, Vancouver BC, Canada; 4 Department of Children's Neurosciences, Evelina London Children's Hospital at Guy's and St Thomas’ NHS Foundation Trust, King's Health Partners Academic Health Science Centre, London, UK; 5 Department of Radiology, Great Ormond Street Hospital for Children, London, UK; 6 Institute of Child Health, University College London, London, UK; 7 The Encephalitis Society, Malton, North Yorkshire, UK; 8 Psychological services (Paediatrics), Alder Hey Children's NHS Foundation Trust, Liverpool, UK; 9 Institute of Infection and Global Health, University of Liverpool, Liverpool, UK; 10 Littlewoods Neuroscience Foundation, Department of Neurology, Alder Hey Children's NHS Foundation Trust, Liverpool, UK; 11 Department of Paediatric Neurology, Oxford University Hospitals NHS Trust, Oxford, UK; 12 National Institute for Health Research Health Protection Research Unit in Emerging and Zoonotic Infections, University of Liverpool, Liverpool, UK; 13 Walton Centre NHS Foundation Trust, Liverpool, UK; 14 Department of Clinical Neurosciences, Weatherall Institute of Molecular Medicine, University of Oxford, Oxford, UK; 15 Nuffield Department of Primary Care Health Sciences, University of Oxford, Oxford, UK

**Keywords:** ADEM, autoimmune, encephalitides, immune-mediated, GOSE-Peds

## Abstract

**Introduction:**

Infectious and immune-mediated encephalitides are important but under-recognised causes of morbidity and mortality in childhood, with a 7% death rate and up to 50% morbidity after prolonged follow-up. There is a theoretical basis for ameliorating the immune response with intravenous immunoglobulin (IVIG), which is supported by empirical evidence of a beneficial response following its use in the treatment of viral and autoimmune encephalitis. In immune-mediated encephalitis, IVIG is often used after a delay (by weeks in some cases), while diagnosis is confirmed. Wider use of IVIG in infectious encephalitis and earlier use in immune-mediated encephalitis could improve outcomes for these conditions. We describe the protocol for the first ever randomised control trial of IVIG treatment for children with all-cause encephalitis.

**Methods and analysis:**

308 children (6 months to 16 years) with a diagnosis of acute/subacute encephalitis will be recruited in ∼30 UK hospitals and randomised to receive 2 doses (1 g/kg/dose) of either IVIG or matching placebo, in addition to standard treatment. Recruitment will be over a 42-month period and follow-up of each participant will be for 12 months post randomisation. The primary outcome is ‘good recovery’ (score of 2 or lower on the Glasgow Outcome Score Extended—paediatric version), at 12 months after randomisation. Additional secondary neurological measures will be collected at 4–6 weeks after discharge from acute care and at 6 and 12 months after randomisation. Safety, radiological, other autoimmune and tertiary outcomes will also be assessed.

**Ethics and dissemination:**

This trial has been approved by the UK National Research Ethics committee (South Central—Oxford A; REC 14/SC/1416). Current protocol: V4.0 (10/03/2016). The findings will be presented at national and international meetings and conferences and published in peer-reviewed journals.

**Trial registration numbers:**

NCT02308982, EudraCT201400299735 and ISRCTN15791925; Pre-results.

**Strengths and limitations of this study**

- This will be the first randomised controlled trial to evaluate the effect of early intravenous immunoglobulin (IVIG) treatment in encephalitis from any cause in children, aiming to recruit a large sample size (N=308) across 30 hospitals.
- Outcome measures will use robust validated and internationally accepted assessment tools and all trial data will be assessed by blinded investigators.
- The trial is expected to provide data on the role of IVIG in reducing poor outcomes following encephalitis from any cause, which would impact on care pathways and individual patient decisions within the health services community, in the UK and internationally and will also inform on health and social care costs.
- Expected recruitment has been based on the reported UK incidence of encephalitis and a high and consistent recruitment rate is required across all centres due to the low disease incidence. While the trial is expected to recruit well at all sites, it is possible that there could be unexpected under-recruitment at one or more sites which would be a barrier to timely completion.
- Given that patients with all forms of encephalitis will be enrolled to the trial, a statistically significant effect may be masked if there is a benefit from IVIG in only one or some aetiological subgroups.

## Introduction

### Background and rationale

Encephalitis is inflammation of the brain parenchyma and manifests as a clinical syndrome characterised by a combination of encephalopathy, behavioural changes, fever, seizure and focal neurological deficits.^1^ In England, the population incidence for all-cause encephalitis is estimated at 5.23–8.66/100 000/year,^2^ with infants and adults >65 years being the most affected.^2^ Diagnosis is typically made by a combination of clinical, laboratory, neuroimaging and electrophysiological findings using an internationally agreed consensus definition.^1^ ^3^ Infections, usually viral, are the most common cause of acute encephalitis, where the cause is identified. Immune-mediated forms of encephalitis, usually characterised by the detection of neuronal antibodies in serum and/or cerebrospinal fluid (CSF), have been described, although the proportion is not yet clear.^4^ ^5^

Encephalitis causes significant morbidity and mortality with up to 7–20% death rate for certain types^6–8^ and up to 50% of survivors reporting deficits such as memory loss, seizures, learning disability and functional impairment after prolonged follow-up.^9–13^ The significant burden of the disease despite the current standard treatment highlights the need to identify strategies to reduce poor outcomes in patients with encephalitis. Encephalitis also imposes a substantial economic and resource burden on healthcare services. A review of encephalitis admissions to paediatric intensive care units showed an average length of stay of 4.3 days, with 75% of children requiring ventilation, and some requiring cardiovascular support (17%) and renal dialysis (6.5%).(Unpublished observations. Iro MA. A population based observational study of childhood encephalitis in children admitted to paediatric intensive care units in England and Wales). A UK study of encephalitis hospitalisations reported a mean length of stay of 34 days and a cost to the National Health Service of >£40 million/year.^2^

Notwithstanding the aetiology, the common pathophysiological process in infectious and autoimmune encephalitis is brain inflammation. There is evidence that intravenous immunoglobulin (IVIG) has a beneficial role in encephalitis from its therapeutic and prophylactic use in enteroviral encephalitis in the immunocompromised and in outbreaks of enterovirus-71 infections in Asia,^14^ as well as other infectious causes of encephalitis.^15–17^ Acute immune treatment, including IVIG, also appears to benefit adults and children with autoimmune encephalitis.^18^ Randomised controlled trials (RCTs) have demonstrated IVIG efficacy in a number of neurological conditions that share similar underlying inflammatory mechanisms to encephalitis even if different aetiologies.^19^ IVIG appears to inhibit complement binding, neutralise pathogenic cytokines, downregulate antibody production and modulate phagocytosis and T-cell function.^20^

In clinical practice, the use of IVIG in encephalitis varies. In the immune-mediated forms of encephalitis, IVIG is often used after a period of delay (by weeks in some cases), while the diagnosis is being confirmed. In other cases, IVIG is used as a last treatment option, usually after several days from hospital admission, where clinical improvement is slow. This delay may limit its benefit due to the brain inflammation, which has already occurred. The variation in practice is due to a lack of class 1 evidence to support the use of IVIG in encephalitis and it is currently unknown whether the wider use of IVIG in infectious encephalitis and earlier use in immune-mediated encephalitis could alter the outcome of this group of conditions. There is therefore the need to fill this evidence gap.

At present, there are no robust controlled trials in children to inform on the optimal treatment of encephalitis. Given the available evidence of possible beneficial role of IVIG, it is therefore important to undertake a trial to investigate the effect of IVIG for all children presenting with encephalitis, and optimise the use of this expensive and limited resource.

### Trial objectives and design

The *I*mmuno*g*lobuli*N i*n the *T*reatment of *E*ncephalitis (IgNiTE) trial is a multicentre, double-blind, placebo-controlled, parallel arm, RCT that will evaluate whether early treatment with IVIG provides benefit for children with a diagnosis of encephalitis, when compared with standard therapy alone. In the context of the IgNiTE trial, ‘early treatment’ is defined as administration of IVIG within 120 hours from presentation to any hospital or, for transferred patients, within 72 hours from admission to a recruiting hospital even if >120 hours since initial hospital presentation.

It is expected that the IgNiTE trial will generate first class evidence to inform clinical decisions regarding the use of IVIG for children with acute and subacute forms of infectious and inflammatory encephalitis.

### Primary objective

The primary objective is to compare neurological outcomes of children with encephalitis who have been treated with either IVIG or placebo, in addition to standard therapy.

### Secondary objectives

The secondary objectives are

- to compare (1) clinical and (2) further neurological outcomes of children with encephalitis who have been treated with IVIG or placebo, in addition to standard therapy,
- to confirm the safety of IVIG treatment for children with encephalitis,
- to identify the proportion of children with immune-mediated encephalitis,
- to determine the effect of IVIG treatment on neuronal antibody levels in children with immune-mediated encephalitis.

### Tertiary objectives

The tertiary objectives are

- to explore clinically relevant neuroimaging predictors of childhood encephalitis,
- to explore predictors of neurological outcomes in children with encephalitis,
- to explore radiological patterns associated with different types of encephalitis,
- to understand the host inflammatory pathways in encephalitis and the relationship with clinical parameters and the effect of IVIG treatment on these pathways.

## Methods

### Trial setting

The trial is planned to be conducted in ∼30 UK hospitals (tertiary and district general) (see online supplementary table S1).

10.1136/bmjopen-2016-012356.supp1 supplementary tables

### Eligibility criteria

*Inclusion criteria (based on the International Encephalitis Consortium consensus case definition)*^1^

1. Age 6 weeks to 16 years old,
2. Acute (within 24 hours) or subacute (24 hours to 4 weeks) onset of altered mental state (reduced or altered conscious level, irritability, altered personality or behaviour, lethargy) not attributable to a metabolic cause,
3. At least two of:
  - fever >38°C within 72 hours before or after presentation to hospital,
  - new or acute onset brain imaging consistent with encephalitis or immune-mediated encephalopathy,
  - CSF white cell count (WCC) >4/microlitre,
  - generalised or partial seizures not fully attributable to a pre-existing seizure disorder,
  - new-onset focal neurological signs (including movement disorders) for >6 hours,
  - EEG abnormality that is consistent with encephalitis and not clearly attributable to another cause
    and
4. Parent/guardian/legal representative consent to the patient participating in the trial.

Exclusion criteria

The patient will not be enrolled to the trial if any of the following apply, in addition to failure to meet all the inclusion criteria:

- high clinical suspicion of bacterial meningitis or TB meningitis (eg, presence of frankly purulent CSF; CSF WCC >1000/microlitre; bacteria on Gram stain and/or culture),
- prior receipt of any IVIG product during the index admission,
- traumatic brain injury,
- known metabolic encephalopathy,
- toxic encephalopathy,
- hypertensive encephalopathy/posterior reversible encephalopathy syndrome,
- pre-existing demyelinating disorder; pre-existing antibody-mediated central nervous system (CNS) disorder; pre-existing CSF diversion,
- ischaemic or haemorrhagic stroke,
- children with a contraindication to IVIG or albumin,
- known hypercoagulable state,
- significant renal impairment defined as GFR of 29 ml/min/1.73 m^2^ and below (Chronic Kidney Disease Stage 4),
- known hyperprolinaemia,
- known to be pregnant,
- any other significant disease or disorder which, in the opinion of the investigator, may either put the participants at risk because of participation in the trial or may influence the result of the trial, or the participant's ability to participate in the trial,
- participants who are being actively followed up in another research trial involving an investigational medicinal product which has a potential immunomodulatory or neuroprotective effect,
- administration of trial treatment not feasible within the study timeline (120 hours from presentation to any hospital or, for transferred patients, 72 hours from admission to a recruiting hospital even if this is >120 hours from presentation to initial hospital) as determined by the trial team,
- any other condition which, in the opinion of the investigator, may interfere with the ability to fulfil trial requirements, especially relating to the primary objective of the trial (this includes plans to be outside the UK for more than 12 months after enrolment).

In addition, any patient who, in the judgement of the clinician and prior to enrolment, is thought will benefit from IVIG will not be enrolled.

### Interventions

Participants will be randomised to receive two doses of either human immunoglobulin (intervention group) or placebo (control group), in addition to standard therapy (see Methods: assignment of intervention). There will be no set trial definition of standard therapy and this may vary between hospitals since there are currently no established national clinical care pathways for these. Participants will receive 1 g/kg/dose, in weight-based dosing bands (see online supplementary table S2). The IVIG product is Privigen (CSL Behring), supplied in unlabelled as 10 g/100 mL vials. Privigen is a licensed product, further details are outlined in the Product Information^21^ and the Summary of Product Characteristics (https://www.medicines.org.uk/emc/medicine/21359). The placebo is 0.1% Human Albumin Solution (HAS) in 0.9% Sodium Chloride solution which will be manufactured in the Aseptic Production Unit (APU), Pharmacy department, Royal Liverpool and Broadgreen Hospital, Liverpool, UK under cGMP conditions, under its MIA (IMP) licence and also supplied as 100 mL vials. The placebo has been constituted using HAS so as to prevent unblinding.

Packaging and labelling of both trial treatments will also take place at the same location. Labelling, which is identical for both trial treatments, has been approved by the Medicines and Healthcare products Regulatory Authority (MHRA) and conform to Annexe 13 of Good Manufacturing Practice standards and Article 13.3 of Directive 2001/20/EC (http://ec.europa.eu/health/files/eudralex/vol-4/2009_06_annex13.pdf). The APU will provide Qualified Persons services and distribute both trial treatments to the Clinical Trials Pharmacy at each recruiting site where they will be stored under controlled conditions and from where they will be dispensed.

The trial treatment will be prescribed on the participant's drug chart by a clinician who has been delegated for this task and using the suggested wording ‘Immunoglobulin/Placebo for the IgNiTE trial’. In addition, a clinical trials prescription form will be completed. For effective management of the trial treatment stock, and to minimise wastage, individual doses may vary slightly. A dosing guide for participants ≥13.5 kg is provided in a Clinical Study plan and is shown in online supplementary table S2. Participants <13.5 kg will receive 1 g/kg, rounded to the nearest whole gram.

Both trial treatments will be administered intravenously by a nurse who has received relevant trial-specific and Good Clinical Practice (GCP) training, is trained to give intravenous infusions and trained in the recognition and treatment of anaphylaxis. The first dose will be given as soon as possible after enrolment, within the defined timelines (see the Trial objectives and design section). The second dose will be given 24–36 hours after the first dose. The administration rate for the trial treatment will be in line with the guidance outlined in the Summary of Product Characteristics (SmPc) for Privigen and local hospital practices for Privigen administration.

Blood and CSF samples will be obtained before and after administration of the trial treatment (see the Data collection methods section).

### Coenrolment

Participants in the IgNiTE trial may be coenrolled to another study where:

- the study does not involve an investigational medicinal product (IMP),
- the study involves an IMP, which is not thought to have a potential immunomodulatory, or neuroprotective effect, as judged by the investigator.

Patients on the following treatment(s) may not be enrolled to the IgNiTE trial:

- Long-term maintenance immunotherapy (defined as 14 days or more) or within 3 months of stopping. This includes (but not limited to) the following: steroids (>1 mg/kg/day), azathioprine, mycophenolate mofetil, methotrexate, monoclonal anti-inflammatory treatment, for example, rituximab, infliximab (or within 1 year of discontinuing such treatment).

### Outcomes

There are currently no established European core outcomes for encephalitis or acquired brain injury in existence (COMET Initiative website: http://www.cometinitiative.org, searched 22 February 2016). The selected outcome measures reflect recommendations by The American Academy of Neurology Common Data Elements Project for neurological assessment post traumatic brain injury in children (accessible @ http://www.commondataelements.ninds.nih.gov). The secondary outcome measures will support the data obtained from the primary outcome.

#### Primary outcome

The primary efficacy outcome is ‘good recovery’, defined as a score of 2 or lower on the Paediatric version of the Glasgow Outcome Score-Extended (GOSE-Peds), at 12 months after randomisation. The GOS-E Peds is a modified version of the GOSE, a gold standard for measuring traumatic brain injury outcome in adults. The GOS-E Peds provides a developmentally appropriate structured interview necessary to evaluate children across different age groups, and it provides a valid measure of outcome in infants, toddlers, children and adolescents. Its use has been validated and found to be sensitive to severity of injury and to recovery over time, at least 6 months after brain injury and has been suggested as useful in guiding treatment in the early phases of recovery from brain injury.^22^ A strong correlation is also seen with parent report of functional outcomes and also with most performance-based cognitive tests for younger and older children. A 6-month assessment has also been chosen (see secondary objectives) as this has the advantage of improved trial retention, and earlier impact assessment.

#### Secondary and tertiary outcomes

These are outlined in table 1.

**Table 1:** Secondary and tertiary outcomes

Data collection time point	Outcome measure
*Secondary outcomes*	
Clinical and neurological	
During hospital inpatient stay	Glasgow coma score Neurological examination findings as documented by the clinical team Duration of invasive ventilation (if ventilated) Length of intensive care unit (ICU) stay in a subset of children admitted to ICU Length of hospitalisation
Around 4–6 weeks after discharge from acute care	Strength and Difficulties Questionnaire (SDQ) Adaptive Behavior Assessment System-Second Edition (ABAS-II) Peds Quality of Life scoring algorithm Liverpool Outcome Score Gross Motor Function Classification System (GMFCS)
Around 6 months (±4 weeks) after randomisation	GOSE-Peds
Around 12 months (±4 weeks) after randomisation	New diagnosis of epilepsy Use of antiepileptic treatment Strength and Difficulties Questionnaire (SDQ) Adaptive Behavior Assessment System-Second Edition (ABAS-II) Peds Quality of Life (PedsQoL) scoring algorithm Liverpool Outcome Score (LOS) Gross Motor Function Classification System (GMFCS) Blinded neuropsychologist assessment of cognitive functioning using age appropriate developmental scales (Bayley Scales for Infant Development (BSID-III)/Wechsler preschool and Primary Scale of Intelligence III (WPPSI-III)/Wechsler Intelligence Scale for Children IV (WISC-IV)
12 months after randomisation	Proportion of deaths occurring in participants
Radiological	
Around 6 months after randomisation	Brain MRI to assess lesion resolution, presence of new lesions and distribution of persisting disease
Safety	
24–48 hours after the second IMP dose	Full blood count check to monitor for haemolysis
First 5 days after each dose of trial treatment	Adverse events of special interest (AESIs)
Up to 6 months after randomisation	Serious adverse events (SAEs)
Up to 12 months after randomisation	Serious adverse reactions (SARs)Suspected Unexpected Serious Adverse Reactions (SUSARs)
Autoimmune	Presence of and comparison of levels of specific neuronal antibodies in serum and/or CSF samples (where lumbar puncture is performed as part of routine care) before and after administration of trial treatment
*Tertiary outcomes*	Correlate MRI findings with neurological outcomes Correlate clinical and laboratory parameters with neurological outcomes Comparison of brain MRI findings with aetiological diagnosis Identification of specific DNA sequence and structural genetic variants in patients with encephalitis The following will be assessed before and after receipt of trial treatment: Comparison of inflammatory cytokines Assessment of regulatory T-cell frequency and function in blood and/or CSF Measurement of inflammatory markers in blood and/or CSF Analysis of gene expression in whole blood Comparison of the host inflammatory pathways and correlation with clinical parameters

### Participant timeline

Time schedule for enrolment, interventions, assessment and visits for participants is shown in table 2.

**Table 2:** Schedule of trial procedures

	T0: As soon as possible after identification of a potential participant and to allow timely administration of trial treatment	T1: As soon as possible after enrolment*	T1+ 24 hours: 24 hours after first dose of trial treatment	T2: 24–36 hours after first dose of trial treatment	T2+ 24–48 hours: 24–48 hours after second dose of trial treatment	T2+7: 7 days after second dose of trial treatment	T3: On the day of discharge from acute care and up to 48 hours prior	T4: 4–6 weeks after discharge from acute care	T5: 6 months (±4 weeks) after randomisation	T6: 12 months (±4 weeks) after randomisation
Eligibility assessment	X									
Informed consent and assent (where appropriate)†	X						X‡		X‡	X‡
Enrolment	X									
Obtain relevant clinical data§	X	X	X	X	X	X	X	X	X	X
Randomisation	X	X ¶								
Scavenged samples§	X	X	X	X	X	X	X	X	X	X
Additional (research sample) where consent is given	X (baseline sample, prior to receipt of trial treatment: neuronal antibody testing, cytokine and DNA analysis, cellular immunology††)	X (where baseline sample not previously obtained and before administration of trial treatment)	X (functional genomics, DNA analysis**)			X (cellular immunology††, functional genomics, DNA analysis**)			X‡‡ (convalescent sample: neuronal antibody testing, cellular immunology†† and cytokine analysis, functional genomics)	
Mandatory full blood count check					X					
Administration of trial treatment and monitoring		X		X						
Completion of Data Capture Form and eCRF§	X	X	X	X	X	X	X	X	X	X
Adverse event assessment (AESIs, SARs, SUSARs and SAEs)		X	X	X	X	X	X	X	X	X§§
Questionnaire completion (ABAS-II, SDQ, GMFCS, Peds QL)								X	X	X
Liverpool Outcome Score								X		X
GOSE-Peds									X	X¶¶
Research MRI (where consent is given)									X***	
Neuropsychology assessment										X

*Visit must be 120 hours from presentation to any hospital OR, for transferred patients, 72 hours from admission to a recruiting hospital even if >120 hours has elapsed since presentation to the initial (referring) hospital.

†Participant consent if 16 years and assent if <16 years.

‡Where consent/assent (as appropriate) has not been previously obtained.

§Continuous process throughout the study.

¶First dose of trial treatment may be given on the same day as randomisation.

**Where DNA sample not previously obtained. Only one DNA sample is required.

††Selected centres only.

‡‡To avoid an extra visit solely for this purpose, the ‘6-month research sample’ can be obtained at any routine follow-up clinical appointments that occur after the participant has been discharged from acute care.

§§Only deaths or where a serious adverse event is judged to be directly related to the trial treatment.

¶¶Primary outcome measure.

***Where consent obtained. May not be required if having routine clinical MRI scan ≥3 months after randomisation.

### Trial duration

The trial is planned to last 5 years which includes a 42 months for recruitment, 12-month follow-up period for each participant and 6 months for data analysis.

### Sample size

There is a near paucity of RCT data from previous studies to estimate sample size for this trial. The sample size calculation is based on the assumption that detection of at least 20% difference from 43% in the ‘good recovery’ rate (ie, GOS-E-Peds score 2 or lower) by 12 months after randomisation is likely to be clinically significant. This is similar to a large observational study on autoimmune encephalitis.^18^ Based on this assumption, a total of 308 participants (154 per group), which takes into account an attrition rate of ∼10%, will provide 90% power and 5% level of significance for a two-sided test.

### Recruitment plan

**Figure 1:**
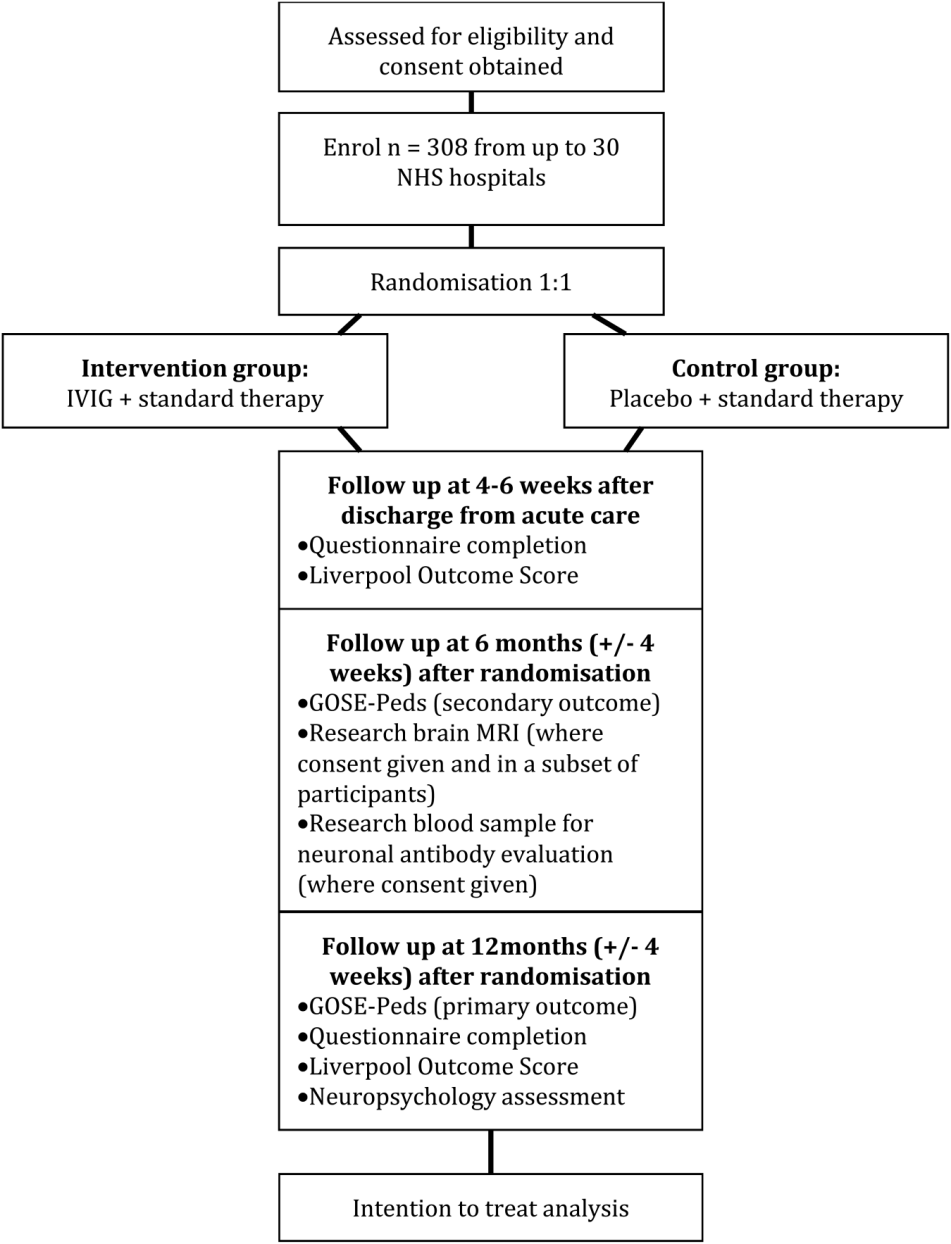
Flow chart showing process of participant recruitment.

A flow chart showing the process of patient recruitment is shown in figure 1. Eligible patients will be identified through various routes: by (1) clinicians reviewing medical handover lists and clinical records of new admissions; (2) research team contacting relevant hospital wards; (3) microbiologists and/or virologists identifying children who have had a lumbar puncture performed for suspected CNS infection; (4) radiologist identifying a brain MRI scan suggestive of encephalitis and (5) neurophysiologist identifying an EEG suggestive of encephalitis.

Following identification of a potential patient through any of the above routes, a member of the clinical team will approach the parent/guardian/legal representative to seek their interest in knowing more about the trial and verbal consent will be sought from the family for their details to be passed on to the trial team. Only if consent for this is granted will a member of the trial team contact the family. A member of the trial team will check the patient's eligibility with the parent/guardian/legal representative, after which they will be provided with the participant information sheet (PIS), if the patient is eligible, and given sufficient time to read this and make a decision regarding participation in the trial. The investigator must obtain informed consent and assent (where applicable and obtainable) before the patient undergoes any trial procedure(s). Once appropriate consent and assent (where applicable and obtainable) have been obtained, the patient will be enrolled to the trial by assigning them a participant number using the next available number from the prepopulated enrolment log.

To maximise achievability of the sample size, we have included mostly tertiary paediatric units that are well placed to recruit rapidly a high number of participants. Potential barriers to recruitment will be identified during the pilot phase of the trial, and close support will be provided to sites with recruitment difficulties. A robust system will be put in place to monitor recruitment to ensure that this is on target. A contingency plan will also be put in place to allow for opening of additional sites in the unlikely event of a less than expected recruitment. A ‘Screening log’ of all screened patients will be kept and will include patients with a diagnosis of encephalitis but are not eligible, eligible patients who refuse to be approached or may not be suitable to be approached, as well as those for whom consent was declined. The reason(s) why a patient is not enrolled will be clearly documented in the screening log, including reasons for declined consent, where this is provided.

### Randomisation

After eligibility is confirmed and consent (and assent where applicable) obtained, enrolled participants will be randomised as soon as possible to allow early administration of the trial treatment in line with the protocol. Randomisation will be performed using a fully validated online randomisation system developed by the Primary Care and Vaccines Collaborative Clinical Trials Unit, University of Oxford, and during working hours when the trial treatment are available. Participants will be randomised in a 1:1 ratio to either an intervention or control group. Only trained research staff with appropriate access and who are on the IgNiTE trial delegation log will be able to randomise patients. The incidence of encephalitis is higher in infants and some forms of encephalitis are more prevalent in certain age groups. In addition, as part of standard care, patients with inflammatory encephalopathy may receive steroid treatment, which may have a beneficial effect. Therefore, to ensure balance between the trial groups, and account for steroid use as confounding variable, randomisation will be stratified by age group n=5 (<1, 1–4, 5–9, 10–14 and 15+ years), and steroid use (yes/no) at the time of enrolment, and using randomly varying block sizes. A computer-generated randomisation code at the time of randomisation will ensure concealment of allocation.

### Withdrawal from trial treatment

The participant will be discontinued from the trial treatment at any time if the investigator considers it necessary for any reason including:

- ineligibility (either arising during the trial or retrospectively having been overlooked at screening),
- significant protocol deviation,
- significant non-compliance with treatment regimen or trial requirements,
- an adverse event (AE) which requires discontinuation of the trial treatment or results in inability to continue to comply with trial procedures,
- disease progression which requires discontinuation of the trial treatment or results in inability to continue to comply with trial procedures.

A participant may also voluntarily withdraw from the trial treatment due to what he or she perceives as an intolerable AE, or for other reasons if they wish.

### Blinding

IgNiTE is a double-blind trial and a rigid blinding process will be in place throughout the trial to ensure validity of the data collected. Participants and their parents/guardians/legal representatives as well as all research staff involved in any aspect of the trial conduct, including recruitment, administration of trial treatment, carrying out trial assessments, data collection and entry, sample and statistical analyses, will be blinded to treatment allocation throughout the entire trial period. There will be separate monitors for blinded and non-blinded data. The active treatment and placebo will be visually identical (packaged and labelled in the same manner) and administered at the same dose and infusion rate to maintain blinding. To be able to manage trial treatment stock effectively and minimise wastage, the clinical trials pharmacists at each recruiting site who are independent of the trial will be non-blinded. The tear off section of the label will inform the dispensing pharmacist of the true nature of the contents (IVIG or placebo). At dispending, this section of the label will be removed to maintain blinding.

Unblinding of treatment allocation will occur only in exceptional circumstances when knowledge of the actual treatment received is absolutely essential for further management of the participant. Unblinding will be performed via the online randomisation system. The decision to unblind a participant's treatment allocation will be solely that of the site investigator. Only individuals given access to unblind will be able to do this and will include the site pharmacist, principal investigator and coinvestigators. Where there is a problem with Sortition, unblinding will be available via either the site pharmacist (during work hours) or a non-blinded staff member in Oxford (out of hours) who is independent of the trial, both of whom will have secure access to the master randomisation list for this purpose.

### Data collection methods

Trial data will be collected by delegated research staff with appropriate training using two methods: (1) a paper-data capture form and (2) an electronic case report form (CRF), OpenClinica which is a password-protected, web-based database with accountability records that is stored on a secure sever within the UK. Trial data will be obtained from various sources, including patient medical notes, parent interview, laboratory reports, brain scan pictures and reports, EEG reports, pharmacy records, drug charts, questionnaires and any correspondences relating to the participants involvement in the trial.

Different data types will be collected throughout the trial period.

#### Clinical data

These will include information regarding patient demographics, clinical findings, treatment, investigation results, length of hospital stay and intensive care management. These data will be obtained throughout the participant's time in the trial (table 1).

#### Questionnaires and outcome measures

Validated questionnaires assessing behaviour, motor and adaptive functioning and quality of life will be completed by the participant and/or by their parent/guardian/authorised legal representative at: (1) 4–6 weeks following discharge from acute care and (2) 12 months after randomisation:

1. Adaptive Behavior Assessment System, second edition,^23^ ^24^
2. Gross Motor Function Classification System,^25^
3. Strength and Difficulties Questionnaire,^26^
4. Paediatric Quality of Life Inventory.^27^ ^28^

Outcome scores will be assessed using the (1) Paediatric version of the Glasgow Outcome Score Extended (GOSE Peds) at 6 and 12 months after randomisation and (2) Liverpool Outcome Score (LOS) at 4–6 weeks after discharge from acute care and 12 months after randomisation.

Various measures to obtain complete follow-up data will be implemented: (1) blinded research staff or the participant's clinician can assist with questionnaires, (2) prepaid envelopes will be provided for return of questionnaires and families will be reminded by telephone, post and/or email and (3) the primary outcome (GOSE Peds) assessment will be completed by the neuropsychologist at the 12-month visit. The neuropsychologist may also assist with questionnaire completion.

#### Laboratory data

Blood and CSF samples (only obtained at the same time as routine lumbar puncture) will be obtained from participants at different time points for neuronal antibodies, cytokine, functional genomics, DNA and cellular immunology evaluation are optional (table 2). A mandatory blood sample will be obtained at 24–48 hours following the second dose of trial treatment to assess full blood count levels as a risk mitigation measure to monitor for haemolysis, which is a reported side effect of high-dose IVIG treatment. Blood sample volumes will be in line with the Medicines for Children Research Network recommendation.^29^ CSF volumes will be in line with British Infection Society TB guideline.^30^ All samples will be anonymised. A sample collection and processing guide will be made available to all recruiting sites.

#### Radiological data

All brain scans (and reports) performed as part of routine clinical care during the study period will be collected. An optional research MRI scan will be performed around 6 months after randomisation for participants who consent to this and where a routine follow-up clinical scan is not being performed. Where a clinical scan is planned for ≥3 months after randomisation, this will be used instead and a research MRI scan will not be necessary. All scans will be anonymised and sent to Great Ormond Street Hospital, London, for analyses by a blinded study neuroradiologist and imaging scientist. MRI findings will be correlated with the primary neurological outcome assessed at 12 months postrandomisation. Further exploratory correlations with other neurological outcomes assessed at the different study time points will also be performed.

#### Neuropsychology assessment

This will be performed at 12 months after randomisation by a blinded trial neuropsychologist using age appropriate, validated scales of developmental assessment (see table 1).

#### Adverse events

Information on AEs will be collected throughout the trial (see the Harms section).

### Withdrawal

Participants may withdraw from the trial at any time. No further data will be collected. Data collected up until the point of withdrawal from the trial will be analysed unless the parent/participant decides against this. If a participant is withdrawn due to an AE, the investigator will follow this up until it resolves or stabilises. All participants who are withdrawn from trial treatment (see the Withdrawal from trial treatment section) will remain in the trial and followed up as per the trial protocol, but will not have any invasive procedures performed. The trial data will be analysed on an intention-to-treat (ITT) basis; therefore, all withdrawals, either from the trial or from the trial treatment, will be reported and included in the data analyses. All protocol deviations will also be reported.

### Study data

#### Data management

Data management will be via the OpenClinica database. All relevant data recorded elsewhere (see data collection methods) that are required to achieve the trial objectives will be transferred on to the OpenClinica database from where they can be downloaded for analysis. To maintain a high-quality standard of data entry, the database will be tested and validated prior to use. In addition, research staff will receive appropriate level training on data collection and entry and there will be regular monitoring of trial data throughout the trial. Furthermore, prior to data analysis, the database will be locked for cleaning to ensure that data are complete and reliable. Research staff at the various recruiting sites will be contacted to provide information on any missing data and to clarify any errors identified. All trial documents will be retained and stored securely in accordance with GCP after the completion or discontinuation of the trial for 3 years after the youngest participant turns 18 years.

### Statistical methods

The primary statistical analysis will be carried out on the basis of ITT. After randomisation, participants will be analysed according to their allocated treatment group irrespective of what treatment they actually receive. However, a further modified ITT analysis will be performed excluding participants found to be ineligible in retrospect.

Data analysis will be performed using a mixed effect model for repeated measures, that is, to incorporate all outcome data collected during the 12 months follow-up, in order to apply the ITT principle as far as possible and to account for potential biases arising from loss to follow-up. The model will include treatment group, time, treatment-by-time interaction and baseline covariates. An unstructured correlation matrix will be used to model the within-participant error correlation structure. An appropriate contrast will be specified to test for treatment efficacy between randomised groups at 12 months. Various sensitivity analyses will be performed using other imputation methods, as well as analysis of 12-month data cross-sectionally, to test whether the results are robust to different assumptions about the missing data. The primary ITT analysis will account for steroid use before randomisation as a covariate. As required, the impact of posthospitalisation course including the use of concomitant and/or different immune treatments and period of neurorehabilitation on the primary outcome will be investigated in an exploratory analysis.

The results from the trial will be prepared as comparative summary statistics (difference in response rate or means) with 95% CIs. All the tests will be performed at a 5% two-sided significance level. A full detailed analysis plan (including plans for any interim analysis, subgroup analysis and sensitivity analysis) will be prepared and finalised before the first interim analysis.

#### Primary analysis

The primary efficacy end point in this trial is ‘good recovery’, defined by GOS-E-Peds score 2 or lower, at 12 months from randomisation. This will be analysed using a generalised linear mixed effect model, using data collected at discharge, 6 and 12 months from randomisation. An interaction between time and randomised group will be fitted to allow estimation of treatment effect at each time point. The model will adjust for baseline values and other stratification factors (eg, age and steroid treatment at the time of randomisation).

#### Secondary and tertiary analyses

As far as possible, we will use similar method for secondary and tertiary continuous outcomes collected at multiple time points or analysis of covariance for those collected at 12 months only, adjusting for baseline measures (if collected) and any stratification variables. Otherwise, an equivalent non-parametric method will be used for outcomes that violate the normal distribution assumption. A log-binomial regression will be performed on binary outcomes with similar adjustment of baseline covariates. χ^2^ or Fisher's exact test will be used to analyse AEs and non-adherence.

Reporting of the trial findings will be in line with Consolidated Standards of Reporting Trials (CONSORT) guidelines.

### Interim analysis

Analysis for the DSMC will be performed in accordance with the DSMC Charter. No interim efficacy analysis will be performed. Interim reports containing safety data, along with any other analyses that the committee may request, will be sent to the DSMC in strict confidence. Close monitoring to assess practical aspects of delivering the trial interventions and recruitment will also be undertaken.

### Data monitoring

The Data Safety Monitoring Committee (DSMC) is responsible for safeguarding the interests of trial patients, monitoring the accumulating data and making recommendations to the Trial Steering Committee (TSC) on whether the trial should continue as planned. The DSMC will comprise of a clinical chair, clinicians and a statistician, all of whom will be independent of the trial, the sponsor and funders. The role of the TSC is to provide overall supervision for the IgNiTE trial on behalf of the Trial Sponsor and the Trial Funder and to ensure that the IgNiTE trial is conducted according to the guidelines for GCP, Research Governance Framework for Health and Social Care and all relevant regulations and local policies. The TSC will comprise an independent chair, the chief investigator (CI), paediatricians and patient representatives. In discharging its safety role, the TSC will work in conjunction with DSMC for the IgNiTE trial. DSMC and TSC will meet prior to trial start and 6 months thereafter. Increased frequency of meetings will be arranged depending on the requirements of the trial, DSMC and TSC recommendations.

### Stopping guidelines

This trial may be suspended or prematurely terminated by the sponsor, CI, regulatory authority or funder if there is sufficient reason to think that the safety of participants is affected by the trial procedures. Written notification, documenting the reason for trial suspension or termination, will be provided by the suspending or terminating party to the investigator, funders and regulatory authorities. If the trial is prematurely terminated or suspended, the CI will promptly inform the REC, MHRA and CSL Behring and will provide the reason(s) for the termination or suspension.

### Harms

International Conference on Harmonisation (ICH) definitions are used for AEs, adverse events of special interest (AESIs), adverse reactions (ARs), serious AEs (SAEs), serious adverse reactions (SARs) and suspected unexpected SARs (SUSARs). IVIG has a well-established side-effect profile. All participants will be monitored for (1) AESIs (includes anaphylaxis, haemolysis, new-onset seizure or abnormal movements not thought to be due to the encephalitis illness, thromboembolism, aseptic meningitis unrelated to the encephalitis illness, acute renal failure and any other medically significant events as determined by the investigator), in the first 5 days following receipt of trial treatment, (2) SAEs up to 6 months after randomisation, or up to 12 months after randomisation where the event is judged directly related to the trial treatment and (3) deaths up to 12 months after randomisation.

Monitoring and reporting of AEs will be performed by the site PI and research team, and will be recorded on the data capture form and uploaded to the eCRF (OpenClinica). The nature and severity of each AE, and the relationship to trial treatment will be documented. The expectedness of an AE will be determined by whether or not it is listed in the SmPC for Privigen or Human Albumin Solution. AESIs and SAEs will be reported to the CI and CSL Behring within 24 hours of the research staff becoming aware. The CI will notify the DSMC of all AESIs and SAEs. This will be expedited (within 24 hours of the CI becoming aware), for all AESIs and for all SAEs that are judged related to the trial treatment. Those that are judged to be unrelated to the trial treatment do not require expedited reporting to the DSMC.

The CI will report all relevant information about a suspected unexpected adverse reaction (SUSAR) that occurs during the course of the trial to the MHRA, CSL Behring, the relevant ethics committee and the DSMC. For fatal and life-threatening SUSARS, this will be performed no later than 7 calendar days after the Sponsor or delegate is first aware of the reaction. Any additional relevant information will be reported within 8 calendar days of the initial report. All other SUSARs will be reported within 15 calendar days. The CI or delegate will also inform all principal investigators concerned of relevant information about SARs that could adversely affect the safety of participants.

A summary list of all SAEs (including those unrelated to the trial treatment), AESIs and SUSARs will be provided in a safety report to the DSMC, which will be submitted at regular interval as specified in the DSMC Charter. In addition, a strict data sheet will be kept which will include the randomisation code aligned to the batch number of assigned IVIG product and in order to maintain a link between the participant and the batch of the product.

### Pregnancy

Although not AEs, pregnancies are reportable events. Should a participant become pregnant during the trial, the trial treatment will be discontinued. Any pregnancy occurring during the clinical trial will be reported to the CI and CSL Behring within 24 hours of the investigator becoming aware and will be followed up for an outcome, which will be recorded. If a congenital abnormality or birth defect is identified, this would fall within the definition of an SAE and will be reported as such.

### Auditing

Regular monitoring by the trial sponsor or delegate will ensure compliance with GCP. The investigator sites will provide direct access to all trial-related source data/documents and reports for the purpose of monitoring and auditing by the sponsor and inspection by local and regulatory authorities. Data will be evaluated for compliance with the protocol and accuracy in relation to source documents. Following written standard operating procedures, the monitors will verify that the clinical trial is conducted and data are generated, documented and reported in compliance with the protocol, GCP and the applicable regulatory requirements. The Quality Assurance manager will also maintain an internal audit programme, which will supplement the external monitoring process to ensure that systems relating to trial conduct, data recording, analysis and reporting are functional are in compliance with the protocol, GCP and the applicable regulatory requirements. The audit programme also includes laboratory activities taking into consideration the MHRA and EMA guidelines for GCP in the laboratory. The Sponsor may carry out audit to ensure compliance with the protocol, GCP and appropriate regulations. GCP inspections may also be undertaken by the MHRA to ensure compliance with protocol and the Medicines for Human Use (Clinical Trials) Regulations 2004.

## Ethics and dissemination

### Ethical and safety considerations

This trial has been approved by the UK National Research Ethics Service (NRES) committee (South Central—Oxford A; REC 14/SC/1416). Clinical trial authorisation has been granted via the Medicines and Healthcare Products Regulatory Agency (MHRA) notification scheme (Ref: 21584/0337/001-0001). Current protocol: V4.0 (10/03/2016). Written approval from the respective Research and Development (R&D) departments will be obtained for each participating site prior to recruitment.

The CI will ensure that this trial (and all subsequent approved amendments) is conducted in accordance with the principles of the Declaration of Helsinki (1996), in full conformity with the International Conference on Harmonisation of Technical Requirements for Registration of Pharmaceuticals for Human Use (ICH) Guidelines for GCP (CPMP/ICH/135/95 July 1996), the Research Governance Framework, and the Medicines for Human Use (Clinical Trial) Regulations 2004. The CI will monitor pharmacovigilance and will report to the Research Ethics Committee (REC), MHRA and funders during and at the end of the trial. All protocol modifications will be disseminated to all relevant parties. The findings of the trial will be presented at national and international meetings and conferences and published in peer-reviewed journals.

### Informed consent and assent

Following identification of a potentially eligible participant by the clinical team, a PIS explaining the trial (including the rationale, aims and objectives, treatment assignation), potential risks and benefits, and all the trial procedures will be provided. Parents/guardians/legal representatives or patients, where appropriate (ie, if the patient has capacity), will be allowed sufficient time to consider the information in the PIS, to seek independent advice and to consider participation in the trial. Informed consent (patients aged 16 years and above) and assent (patients below 16 years) will be obtained by trained research staff using an appropriately signed and dated informed consent/assent form, before any trial-specific procedures are performed. Given that children with encephalitis will be unwell and may be confused during the acute illness, it is likely that eligible patients would be unable to provide consent/assent prior to enrolment. Therefore, for patients aged 16 years and above, informed consent will be obtained from their parent/guardian/legal representative. Once capacity is regained, appropriate consent/assent will be sought from all participants at follow-up time points and if this is not granted, they will be withdrawn from the trial. Participants who previously provided assent but turn 16 years while still in the trial will be required to provide consent for ongoing participation in the trial and will be withdrawn if this is not granted.

Parents/guardians/legally authorised representatives/participants may be approached about a separate, ethically approved, Biobank study and asked if they would like to consent to this study using a separate consent form. Participation in the Biobank is optional and samples will only be stored where appropriate consent has been obtained.

### Confidentiality

Data will be stored securely in line with the Data Protection Act 1998. The randomisation system, data capture form and eCRF have been designed so as to protect participant information and to maintain confidentiality. It will be the responsibility of the local investigators to ensure that the data are password protected and held on local trust computer systems. The research staff will ensure that the participants' anonymity is maintained. Participants will be identified only by initials and participant number on the research notes and eCRF. All investigation results and blood samples will be anonymised. All trial documents will be stored securely and only accessible by research staff and authorised personnel. The CI will be the custodian of the trial data.

### Access to data

Direct access will be granted to authorised representatives from the Sponsor or host institution for monitoring and/or audit of the trial to ensure compliance with regulations.

### Reimbursement

Reasonable travel expenses for any visits additional to normal care will be reimbursed on production of receipts, or a mileage allowance provided as appropriate.

### Ancillary and post-trial care

There will be no continued provision of treatment available after participants have completed the trial; however, participants are likely to be followed up by the hospital team as part of routine care. Details of The Encephalitis Society are provided in the PIS, and they can provide ongoing support and information to families.

### Dissemination policy

We aim to produce high-impact publications of the results of the trial and present the findings to the paediatricians who manage encephalitis in the front line. The investigators will be involved in preparing drafts of the manuscripts, abstracts, press releases and any other publications arising from the trial. Authors will acknowledge that the trial was funded by the National Institute for Health Research and CSL Behring. Authorship will be determined in accordance with the International Committee of Medical Journal Editors (ICMJE) guidelines and other contributors will be acknowledged. There is no intended use of professional writers.

### Patient public involvement

The Encephalitis Society provided advice on the clinical problem and need for interventions to address the poor outcomes from encephalitis. The trial proposal was discussed with The Encephalitis Society who affirmed its importance as a priority for evaluation and the Chief Executive of The Encephalitis Society is a coapplicant on the grant application and a coauthor on this paper.

To provide an important patient-centred research perspective, we have engaged members of the public in our PPI programme in the design and management of the trial, through The Encephalitis Society. The opinion of The Encephalitis Society on the burden of the questionnaire outcome measures on patients was sought at the design stage of the trial. The Encephalitis Society also reviewed and provided comments on patient information sheets and consent forms. The Encephalitis Society research poster will be provided for use at the respective recruiting centres. Through The Encephalitis Society, we have also recruited two patient representatives as members of the trial steering committee.

We will provide detailed accessible information about the trial outcomes to patients/parents/carers. The Encephalitis Society will drive forward publication and dissemination of the trial findings among lay, therapeutic and health professionals through the use of web materials, newsletters and guides as well as at conferences and seminars in relation to Encephalitis and related fields. All patients and their parents/carers will be acknowledged in any outputs from the trial. We will also work with The Encephalitis Society on a programme of teaching events and produce guides for healthcare professionals and lay people.
