# Supplementary material for: ImmunoglobuliN in the Treatment of Encephalitis (IgNiTE): protocol for a multicentre randomised controlled trial
Source: BMJ Open. 2016 Nov 3;6(11):e012356. doi: 10.1136/bmjopen-2016-012356 (PMC5129051; doi:10.1136/bmjopen-2016-012356)
Supplement: supplementary tables [file bmjopen-2016-012356supp_tables.pdf]

Supplementary Table 1 List of planned participating sites

1. Addenbrooke's Hospital, Cambridge University Hospitals, Cambridge
2. Alder Hey Children's Hospital NHS Foundation Trust, Liverpool
3. Belfast Hospital for Sick Children, Belfast
4. Birmingham Children's Hospital NHS Foundation Trust, Birmingham
5. Bradford University Hospitals NHS Foundation Trust
6. Evelina Children's Hospital at Guy's and St Thomas' NHS Foundation Trust, London
7. Great Northern Children's Hospital, Newcastle Hospitals NHS Foundation Trust, Newcastle
8. Great Ormond Street Hospital Foundation Trust, London
9. Heartlands Hospital, Heart of England NHS Foundation Trust, Birmingham
10. James Cooke University Hospital, South Tees Hospitals NHS Foundation trust
11. John Radcliffe Hospital, Oxford University Hospitals NHS Trust, Oxford
12. Leeds Teaching Hospital NHS Trust
13. Morriston Hospital, Health in Wales
14. Ninewells Hospital, NHS Tayside
15. Nottingham University Hospitals NHS Trust, Nottingham
16. Pennine Acute Hospital NHS Trust, Manchester
17. Royal Aberdeen Children's Hospital, NHS Grampian (Aberdeen)
18. Royal Cornwall Hospitals NHS Trust
19. Royal Hospital For Children, NHS Greater Glasgow
20. Royal Manchester Children's Hospital, Manchester
21. Sheffield Children's Hospital NHS Foundation Trust
22. St George's University Hospitals NHS Foundation Trust
23. St Mary's Hospital, Imperial College Healthcare NHS Trust
24. University Hospital Bristol NHS Foundation Trust
25. University Hospital of Wales, Cardiff and Vale NHS Trust, Cardiff
26. University Hospitals of Leicester NHS Trust
27. University of Edinburgh, NHS Lothian
28. University of London and Bart's Health NHS Trust, London
29. University Southampton NHS Trust, Southampton

Supplementary Table 2: Dosing guide for trial treatment based on weight band

Weight band (kg)	Dose 1(g)	Dose 2(g)	Total dose (g) to be received by participant
13.5 - 17.4	15	15	30
17.5 - 23.4	20	20	40
23.5 - 27.4	25	25	50
27.5 - 33.4	30	30	60
33.5 - 35.4	35	35	70
35.5 - 45.4	40	40	80
45.5 - 55.4	50	50	100
55.5 - 65.4	60	60	120
65.5 - 75.4	70	70	140
75.5 - 85.4	80	80	160
85.5 - 95.4	90	90	180
95.5 - 105.4	100	100	200
105.5 - 115.4	110	110	220
115.5 - 125.4	120	120	240
